# Differences between intraabdominal candidiasis in regular wards versus intensive care unit

**DOI:** 10.1186/2197-425X-3-S1-A115

**Published:** 2015-10-01

**Authors:** L Lagunes, B Borgatta, M Antonelli, M Bassetti, P Brugnaro, G Dimopoulos, A Diaz-Martin, AL Colombo, R Luzzati, F Menichetti, P Muñoz, M Nucci, I Palacios-Garcia, G Scotton, C Viscoli, M Tumbarello, J Rello

**Affiliations:** Critical Care Department, Vall d'Hebron University Hospital-VHIR´hebron University Hospital, Barcelona, Spain; Vall d'Hebron University Hospital-VHIR´hebron University Hospital, Barcelona, Spain; Sacro Cuore Catholic University, A. Gemelli Hospital, Rome, Italy; Infectious Diseases division, Santa Maria Misericordia University Hospital, Udine, Italy; Infectious Diseases division, Venezia Hospital, Venezia, Italy; Critical Care Department, Attikon University Hospital, Athens, Greece; Virgen del Rocio University Hospital, Seville, Spain; Escola Paulista de Medicina UNIFESP, Sao Paolo, Brazil; University Hospital of Trieste, Trieste, Italy; Azienda Ospedaliera Universitaria Pisana, Pisa, Italy; Hospital General Universitario Gregorio Marañón, Madrid, Spain; Federal University of Rio de Janeiro, University Hospital, Rio de Janeiro, Brazil; Treviso Hospital, Treviso, Italy; Azienda Ospedaliera Universitaria, Genova, Italy; Sacro Cuore Catholic University Hospital, Rome, Italy

## Introduction

Intraabdominal candidiasis (IAC) is a condition associated with a high morbidity and mortality in the Intensive Care Unit (ICU) [1]. Actual guidelines are centered on candidemia [2] and there are uncertainties in the diagnostic criteria for IAC, thus contributing to the scarcity of information.

## Objectives

Describe the characteristics of IAC in ICU patients and assess possible differences between patients with intraabdominal candidiasis in ICU compared to those in regular wards.

## Methods

Retrospective multicenter, multinational study of IAC over 3 years in 13 hospitals in 5 countries. IAC was defined according to a European consensus definitions. Demographic and clinical data was recorded.

## Results

481 patients with intraabdominal candidiasis were recorded. 132 patients (27%) were at the ICU at time of diagnosis and 349 (73%) at regular wards (252 in surgical wards). There were no statistical differences in age, sex or comorbidities except for heart disease (28% ICU vs 15%ward, p= 0,002) and use of dyalisis (11%ICU vs 4% ward p= 0,005). APACHE II score was higher in ICU group (mean:18 IQR 25%-75%:14-24) vs wards (mean: 13 IQR 25%-75%:8-18) p= < 0,001.

Time from hospitalization to diagnosis was longer in ICU group (mean: 13 days IQR 25-75%: 6-30 days) compared to regular ward (mean: 11 days IQR 25-75%: 2-20 days), p = 0,006. Secondary peritonitis was the most common source of infection (39% vs 40% respectively) followed by abdominal abscess, biliary tact infection, pancreatitis and tertiary peritonitis without differences between groups. *Candida albicans* was the most frequent isolated strain in both groups (64% ICU vs 65%ward) follow by *C glabrata, C tropicalis, C parapsilosis* and *C krusei* with no statistical differences. Candida colonization, candidemia and septic shock were more present in the ICU population (40%,23%, and 70% respectively). Echinocandin was initiated in 43,7%, follow by azole in 21% and amphotericin B only 2,7%. Adequate antifungal treatment was more frequent in ICU population (70% versus 56%, p 0,006), adequate source control was similar in both groups (58% and 62% p 0,34) however 30 day mortality was higher in ICU patients ( 39% versus 22% p= < 0,001).

## Conclusions

No differences in source of infection or candida species regarding ICU or regular ward were observed. Adequate initial treatment was higher in the ICU population but patients with intrabdominal candidiasis in the ICU remain as a group with high mortality probably in relation to severity of illness.
